# PARP3 Affects Nucleosome Compaction Regulation

**DOI:** 10.3390/ijms24109042

**Published:** 2023-05-20

**Authors:** Alexander Ukraintsev, Mikhail Kutuzov, Ekaterina Belousova, Marie Joyeau, Victor Golyshev, Alexander Lomzov, Olga Lavrik

**Affiliations:** Institute of Chemical Biology and Fundamental Medicine, Siberian Branch of Russian Academy of Sciences, 630090 Novosibirsk, Russiakutuzov.mm@mail.ru (M.K.);

**Keywords:** nucleosome, atomic force microscopy, poly(ADP-ribose)polymerase, chromatin structure, DNA compaction

## Abstract

Genome compaction is one of the important subject areas for understanding the mechanisms regulating genes’ expression and DNA replication and repair. The basic unit of DNA compaction in the eukaryotic cell is the nucleosome. The main chromatin proteins responsible for DNA compaction have already been identified, but the regulation of chromatin architecture is still extensively studied. Several authors have shown an interaction of ARTD proteins with nucleosomes and proposed that there are changes in the nucleosomes’ structure as a result. In the ARTD family, only PARP1, PARP2, and PARP3 participate in the DNA damage response. Damaged DNA stimulates activation of these PARPs, which use NAD^+^ as a substrate. DNA repair and chromatin compaction need precise regulation with close coordination between them. In this work, we studied the interactions of these three PARPs with nucleosomes by atomic force microscopy, which is a powerful method allowing for direct measurements of geometric characteristics of single molecules. Using this method, we evaluated perturbations in the structure of single nucleosomes after the binding of a PARP. We demonstrated here that PARP3 significantly alters the geometry of nucleosomes, possibly indicating a new function of PARP3 in chromatin compaction regulation.

## 1. Introduction

DNA in eukaryotes is mostly packed into chromatin [1]. The compaction is implemented by chromatin proteins and is presumably regulated by modifications of nitrogenous bases in DNA or chromatin proteins. The compaction level can influence the expression of the affected genes via transcription regulation [2]. The basic unit of DNA compaction is the nucleosome. The main functions of nucleosomes are compaction and the protection of DNA and regulation of gene expression [3]. A nucleosome consists of 147 nt of DNA wrapped around a histone octamer consisting of two molecules of each of the following histones: H2A, H2B, H3, and H4. This nucleoprotein complex is also termed the nucleosome core particle (NCP). The structural and functional details are reviewed in Reference [3].

The higher compaction level is usually mediated by linker histone H1. This histone binds to an NCP in the entry–exit region, thus forming a chromatosome, and chromatosomes can then condense into fibers. This compaction of NCPs requires a certain density of DNA wrapping [4]. Parameters of the fiber may depend on the NCP compaction degree. For example, the replacement of the H3 histone with CenpA leads to less compacted NCPs [5,6]. This alteration probably results in an alternative type of NCP compaction in fibers [5]. Functions of different types of DNA compaction in chromatin are being debated. One of the known chromatin changes occurs in response to the binding of poly(ADP-ribose)polymerase 1 (PARP1) [7].

The diphtheria toxin-like ADP-ribosyltransferase (ARTD) family of proteins consists of 17 members. These proteins share the active site of the catalytic domain [8]. Three proteins from this family—PARP1, PARP2, and PARP3—are known to be DNA-damage-dependent. These PARPs activate in response to DNA damage. They catalyze the transfer of ADP-ribose from NAD^+^ to an acceptor molecule. Various proteins and DNAs can act as an acceptor for these PARPs [9,10,11]. PARP1 and PARP2 can synthesize long branched polymers of ADP-ribose (PAR), whereas PARP3 performs only mono(ADP-ribosyl)ation [12]. PARP1 and PARP2 are regulator proteins in base excision repair and double-strand break repair [13,14,15,16,17]. ADP-ribosylation can perform the function of an intracellular signal for the recruitment of DNA repair proteins. On the other hand, as a type of post-translational modification, ADP-ribosylation influences the properties of a target protein [18].

Several authors have revealed an interaction of the PARP1 protein with NCPs and have proposed that there is a change in the NCP structure as a result [19]. PARP1 can affect the chromatin structure via poly(ADP-ribosyl)ation (PARylation) [20]. Under PARylation conditions, PARP1 modifies histone H1, thus causing its dissociation. Recently, a protein involved in histone PARylation (HPF1) was discovered [21,22]. In the presence of this protein, PARP1 and PARP2 can modify (ADP-ribosyl)ate core histones in an NCP. These modifications lead to chromatin relaxation [23].

PARP1 can also directly affect NCPs. It has been shown that in the absence of linker regions, PARP1′s binding to an end of nucleosomal double-stranded DNA (dsDNA) causes a significant increase in the distance between adjacent gyres of the duplex, and this process is not accompanied by a loss of histones; moreover, it is reversible after PARylation [24]. Such major distortions of the NCP structure may be a consequence of the ability of PARP1 to strongly interact with DNA through the DNA-binding domain (DBD), which includes three Zn-finger domains, a WGR domain, and even a BRCT domain.

Although PARP2 is the closest homolog of PARP1, its DBD is considerably different. PARP2 does not contain any known DNA-binding motifs but comprises a structure similar to the SAP motif. It also has different DNA-binding properties: a lower affinity for free DNA and compacted DNA as compared to PARP1. It has been demonstrated that during the interaction with compacted DNA, PARP2 forms a bridge between two NCPs in double-strand breaks [25]. In contrast to PARP1, the interplay between PARP2 and an NCP has not been described in much detail.

In this regard, PARP3 is less characterized compared to PARP1 and PARP2, and the processes involving PARP3 are being researched at present. PARP3 interacts with PARP1 and several DNA damage repair proteins [26,27,28]. In the cell, PARP3 is reported to be associated with several polycomb group proteins [27]. The latter finding suggests that PARP3 participates in epigenetic regulation of transcription. Notably, this enzyme does not have a structurally separate DBD. The unstructured N-terminus is responsible for this function in PARP3. Nevertheless, PARP3 is strongly activated by DNA strand breaks in vitro and can facilitate non-homologous end joining [27,28,29].

More detailed information about aspects of structural reorganization during direct binding of a PARP protein to an NCP may be obtained by single-molecule methods such as atomic force microscopy (AFM). AFM is an approach used to directly measure the geometric characteristics of individual molecules placed on mica plaque surfaces. It is one of the most widely used nano-tools for studying protein DNA complexes including NCPs [30,31,32,33,34,35,36]. This method can be employed for estimating the NCP compaction degree by measurement of the angle between DNA arms near the entry–exit site of an NCP [31,37]. Using this approach, a stabilizing effect of histone H1 on an NCP has been demonstrated [32].

In our work, we studied the interactions of PARP1, PARP2, and PARP3 with an NCP reconstituted from native core histones and Widom’s clone 603 DNA extended by 79 and 120 bp DNA arms. In particular, we determined changes in the geometric parameters of NCPs during their binding to PARP1, PARP2, or PARP3.

## 2. Results and Discussion

### 2.1. The Localization of PARP Proteins in NCP–PARP Complexes

**Figure 1:**
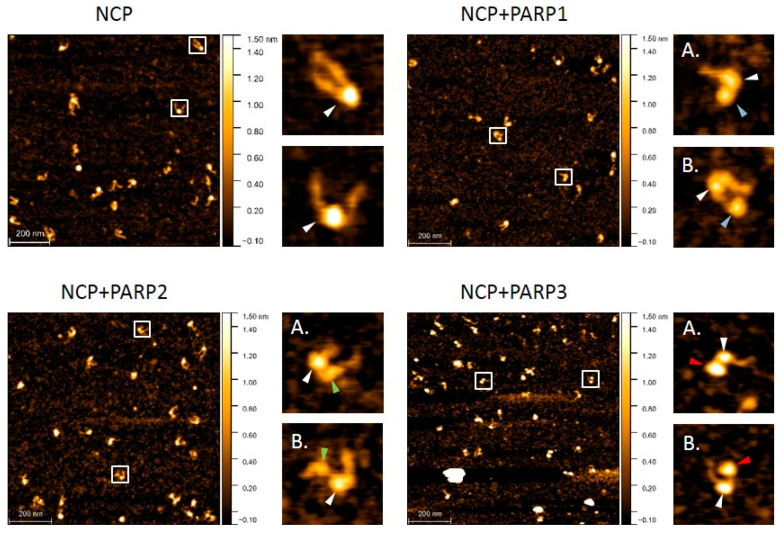
Representative AFM scans of an NCP. Cores of the NCP are indicated by white arrows. PARP1, PARP2, and PARP3 molecules are pointed out by blue, green, and red arrows, respectively. (**A**) A PARP located close to the NCP core. (**B**) A PARP located on the linker DNA region.

First, we determined the site of binding of each PARP protein to our model NCP. For this purpose, reconstituted NCPs were incubated with a PARP followed by immobilization on a mica surface and visualization by AFM scanning in air. Only images of complexes containing both PARP and NCP molecules were chosen for the analysis. According to the positioning of a PARP molecule, the captured images were sorted into two categories: (i) a PARP is located close to the NCP core; (ii) the PARP is located on the linker DNA region. Figure 1 shows typical images of NCPs in their complex with PARPs. While accumulating the data, we found that each of the three PARPs presumably binds near the NCP core: in 153 out of 200 complexes for PARP1, in 148 out of 200 complexes for PARP2, and 158 out of 200 complexes for PARP3.

Strong affinity (in the sub-nanomolar range) of PARP1 and PARP2 for DNA containing various structural elements has been demonstrated earlier [36,38]. In those experiments, naked DNA was used. Additionally, our previous data revealed that K_d_ values are almost identical when complexes of PARP1 with naked DNA and of PARP1 with an NCP are compared [39].

An earlier study uncovered a specific nature of PARP1′s binding to NCP and the ability of this protein to modulate chromatin structure through NAD^+^-dependent automodification without disassembly of the NCP core [40]. These authors also showed that PARP1 is associated with chromatin regions depleted of histone H1. PARP1 saturates chromatin in a molar ratio of 1:1 toward the NCP and competes with H1 for the binding to NCPs. A recent study shows the ability of PARP1 to bind DNA near the entry–exit site of an NCP through the BRCT domain of PARP1 in addition to Zn-finger domains [41]. Furthermore, a condensing effect of PARP1 binding on chromatin has been demonstrated [7]. These data are in agreement with our findings about PARP1 localization during its binding to the model NCP. Taken together, all these data may indicate a potential structural role of PARP1. The binding of PARP1 to an NCP instead of H1 in the absence of DNA damage may lead to a certain temporal pattern of chromatin alterations and to an alternative compaction degree.

Preferential binding of PARP2 to the NCP core was expected here because PARP2 possesses a significantly stronger affinity for NCP compared to naked DNA [39]. The mechanism underlying the interaction of PARP2 or PARP3 with the NCP is not clear, first of all, owing to dramatic differences in the structure of their DBDs from those of PARP1 and differences in subsequent various types of interaction with DNA [42,43]. Moreover, the interaction of PARP2 or PARP3 with the NCP in the absence of DNA damage may be mediated by core histones. In any case, the binding of PARP2 or PARP3 to an NCP may affect its geometry.

### 2.2. The Impact of PARP Binding on the NCP Compaction Degree

Here, we analyzed only the complexes where a PARP molecule is located close to the NCP core. We measured the angle between the linker DNAs of the NCP, i.e., the opening angle (as described in Materials and Methods), to evaluate the changes in the geometric parameters of the NCP. A similar approach was used previously [32,44].

**Figure 2:**
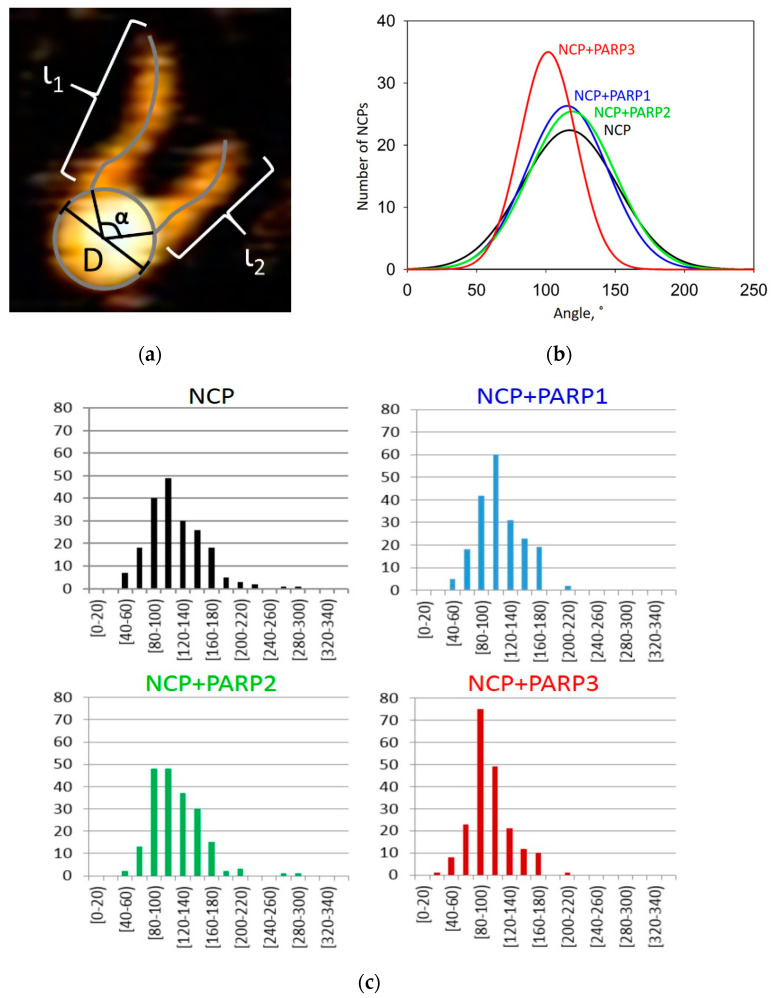
The compaction of NCPs depending on the presence of a PARP protein. (**a**) Schematic representation of determined parameters of an NCP. In the image, “l1” and “l2” are DNA arm lengths, “α” is the angle between DNA arms, and “D” is the diameter of the NCP core. (**b**) The Gauss interpolation of the distribution of “α” angle values. The black curve: NCP samples, the blue curve: samples of NCPs supplemented with PARP1, the green curve: samples of NCPs supplemented with PARP2, and the red curve: samples of NCPs supplemented with PARP3. (**c**) Representation of the distribution of “α” angle values. Black bars: NCP, blue bars: NCP supplemented with PARP1, green bars: NCP supplemented with PARP2, and red bars: NCP supplemented with PARP3.

We analyzed 200 complexes of the NCP under all conditions under study. As a reference sample in the experiment, we utilized an NCP without supplementation with any PARP. Graphical representation of the results is given in Figure 2b. The raw data are presented in Table A1, Table A2, Table A3 and Table A4. The average angle between DNA arms near the entry–exit region for NCPs in native states was estimated as 120° ± 5°. This result is consistent with the data obtained by Jan Lipfert’s group [31]. Those authors showed a dual-mode distribution in 2D density plots, which depicted a correlation between the length of unwrapped DNA and an opening-angle distribution. In contrast to their data, we did not observe such a clear-cut dual-mode distribution in our experiments (Figure A2). This discrepancy may be explained by a difference in the nucleotide sequences of the DNA used. In our work, we employed Widom’s clone 603 DNA (which is characterized by weaker affinity of binding to core histones) instead of clone 601 DNA as used in Reference [31]. The main difference between these two DNA sequences is the toughness of the NCP core: the NCP based on clone 601 DNA is tougher and therefore has less flexible DNA ends. It is probable that our model NCPs based on clone 603 DNA have insufficient differences in their opening-angle values to discriminate clearly between these two modes.

The binding of PARP1 to an NCP caused slight narrowing of the distribution of the opening arms’ angle without a significant effect on the compaction of the NCP (115° ± 4°). The difference in the measured values of the angle in the nucleosome in the presence and in the absence of PARP1 was not significant (even for a *p*-value of 0.9). As mentioned above, PARP1 can bind an NCP near the entry–exit site and interact with both DNA linkers. This interaction can influence the structural functioning of chromatin similarly to linker histone H1. It has been reported that the presence of H1 narrows the opening-angle distribution (meaning NCP stabilization) and does not change the compaction degree [32]. Furthermore, similarly to histone H1, PARP1 compacts chromatin, which is relaxed under PARylation conditions [7]. The authors of Reference [45] propose that the compaction is accomplished via the bringing of neighboring NCPs together by PARP1 molecules, analogously to the process observed in the polycomb group protein complex. This effect is probably due to loop formation caused by PARP1–PARP1 contacts [46]. It should be noted that the binding of PARP1 to DNA near the entry–exit site leads to the distancing of the two DNA gyres, thereby destabilizing the NCP core [24]. Thus, PARP1 loosens the NCP structure. Nonetheless, in that report, the authors demonstrated separation of fluorescent labels located on the DNA helices wrapping the histone core when PARP1 was bound. These data were obtained by the Forster resonance energy transfer technique, which does not discriminate between directions of the NCP deformation; the changes in NCP structure can occur in one of two directions: radial or axial. Because we did not detect significant changes in the compaction degree of the NCP in the presence of PARP1, the changes probably proceed in the axial direction. In this case, the influence cannot be determined by the method under study. We also cannot rule out that the previously described NCP structure distortions caused by PARP1 may affect only the DNA region that is in direct contact with the histone core. In this context, the geometry of the entry–exit site of DNA may be unaltered.

Even though PARP2 manifests significantly stronger affinity for NCPs than for naked DNA, PARP2 (just as PARP1) does not significantly affect the NCP compaction [39]. In the present work, neither the distribution of opening-angle values nor the compaction degree of the NCP was changed by the presence of PARP2 (121° ± 4°). Taking into account the standard deviation, the difference in the measured values of the angle in the nucleosome in the presence and in the absence of PARP2 was not significant (even for a *p*-value of 0.8). In the absence of blunt DNA ends, PARP2 probably binds to NCPs through histones. In this case, it is highly likely that PARP2 mostly binds outside the entry–exit site. Therefore, the impact on the compaction degree may be small.

Meanwhile, PARP3 exerted a distinctive effect on the compaction of the NCP core. We observed an increased compaction degree of the NCPs in the presence of PARP3 (104° ± 4°). Taking into account the standard deviation, the difference in the measured values of the angle in the nucleosome in the presence and in the absence of PARP3 was significant (a *p*-value of 0.001). Moreover, the presence of PARP3 induced the narrowing of the opening-angle distribution. It is worth mentioning that PARP3 is widespread in the nucleus as a part of polycomb group protein complexes. The molecular function of the polycomb group is important for homeotic gene regulation and is consequently suppressed during cell differentiation with the transition of genes into the heterochromatic state. Thus, the effect of PARP3 on NCP compaction may be required for the regulation of the access of other proteins to undamaged DNA via DNA compaction regulation.

In our work, we investigated changes in the NCP architecture during interactions with PARP1, PARP2, or PARP3 in the absence of (ADP-ribosyl)ation. The observed effects can be dramatically altered by the presence of lesions in DNA and NAD^+^. These alterations could also be important, especially because of the different abilities of PARP proteins to synthesize various PAR chains on an acceptor molecule, starting from the transfer of one ADP-ribose (as PARP3 does). What is more, the contribution of accompanying factors such as HPF1 could be substantial during the (ADP-ribosyl)ation and consequent NCP compaction reorganization.

Nevertheless, our study simulates the scenario where DNA is undamaged and the basic ADP-ribose transfer activity of PARPs is weak. To summarize, PARP3 is a new probable player in chromatin compaction regulation.

In conclusion, the clear difference between PARP1, PARP2, and PARP3 in their actions during this process may open up a new research field: the elucidation of PARP3′s function in chromatin compaction in the absence of DNA damage. The question is how to find the conditions (the biological process) where the observed effect is indispensable. The effect may be clarified when higher-order DNA compaction is studied in this context.

## 3. Materials and Methods

### 3.1. Reagents and Equipment

The following reagents and materials were used: 3.5 kDa cutoff dialysis membranes (Spectrum Laboratories Inc., Rancho Dominguez, CA, USA); bromophenol blue and xylene cyanol (Fluka, Buchs, Switzerland). Most of the reagents used in the study were purchased from Sigma (St. Louis, MO, USA). Recombinant Taq DNA polymerase was kindly provided by Prof. Svetlana Khodyreva (Institute of Chemical Biology and Fundamental Medicine, Siberian Branch of Russian Academy of Sciences (ICBFM SB RAS)). Recombinant proteins—human PARP1, murine PARP2, human PARP3, and histone octamers H2A, H2B, H3, and H4 from *G. gallus*—were prepared and isolated as described in References [11,47,48]. AFM imaging was performed on Multimode 8 (Bruker, Billerica, MA, USA) with the help of NSG30_SS probes (TipsNano, Tallinn, Estonia). The synthesis of 1-(3-aminopropyl)-silatrane (APS) was performed as described elsewhere [49]. NCP assembly products were visualized after separation in a polyacrylamide gel by means of a Typhoon FLA 9500 system (GE Healthcare Life Science, Barrington, IL, USA) and Amersham Imager 680 (GE Healthcare Life Science, Barrington, IL, USA).

### 3.2. Preparation of DNA Substrates

The DNA-603-containing substrate used in the experiments was generated by PCR from a pGEM-3z/603 plasmid vector (AddGene, Watertown, MA, USA) with unique primers. The DNA construct contains 147 bp of strong positioning of Widom’s clone 603 DNA sequence surrounded by plasmid DNA sequences of 120 and 79 bp:

5′-GGGCGAATTCGAGCTCGGTACCCGGGGATCCTCTAGAGTCGGGAGCTCGGAACACTATCCGACTGGCACCGAAACGGGTACCCCAGGGACTTGAAGTAATAAGGACGGAGGGCCTCTTTCAACATCGATGCACGGTGGTTAGCCTTGGATTGCGCTCTACCGTGCGCTAAGCGTACTTAGAAGCCCGAGTGACGACTTCACACGGTAGGTGGGCGCGCGAACTGGGCACCCGAGAGTGTCGATTATTTTACGGCTCACGCTGGGGTGATTTGTACTAGGAAAACGCCTATTCGTGTATTCCGCCTTGGTCATTAGGATCCCGGACCTGCAGGCATGCAAGCTTGAG-3′.

Primer oligonucleotides 5′-GGGCGAATTCNAGCTCGGTAC-3′ and 5′-CTCAAGCTTGCATGCCTGCAG-3′ were synthesized in the Laboratory of Biomedical Chemistry at the ICBFM SB RAS (Russia). The following program in PCR was used: 3 min at 94 °C; 30 cycles of 30 s at 94 °C, 20 s at 65 °C, and 1 min at 72 °C; with final extension for 3 min at 72 °C.

After the PCR-based synthesis, the DNA substrate was purified by gel electrophoresis and isolated from the gel by the protocol from reference [50].

### 3.3. NCP Assembly

The NCP assembly was carried out in accordance with our previously described protocol [51]. Briefly, by quick reconstitution of NCPs in analytical amounts, the correct ratio of DNA–histones’ species was determined. Then, preparative reconstitution was performed by gradient dialysis according to the determined ratio.

### 3.4. Preparation of NCP Samples Containing a PARP

Sample preparation for AFM imaging was performed as described before [52]. Freshly cleaved mica was functionalized with a solution of APS for sample deposition.

The reaction mixture was composed of 10 nM NCP, NCP buffer (20 mM NaCl, 0.2 mM EDTA, 1.6 mM CHAPS, 10 mM Tris-HCl pH 7.5, and 5 mM β-mercaptoethanol) and one of PARPs at a concentration of 10 nM (PARP1), 35 nM (PARP2), or 66 nM (PARP3). The reaction mixture was incubated for 15 min at 37 °C. Then, samples were diluted tenfold with Milli-Q water and immediately deposited on the mica surface. After 120 s of deposition, the mica surface was rinsed three times with 1 mL of Milli-Q water and dried in a gentle stream of argon. The samples were stored in a desiccator before the imaging.

### 3.5. AFM Imaging

The visualization was performed in tapping mode in air at a tip resonance frequency of 240–440 kHz. A typical resulting image had a size of 2 µm × 2 µm at 1024 pixels/row or 4 µm × 4 µm at 2048 pixels/row. The scanning rate was either 1.0 or 0.5 Hz, respectively.

### 3.6. Data Analysis

**Figure A1:**
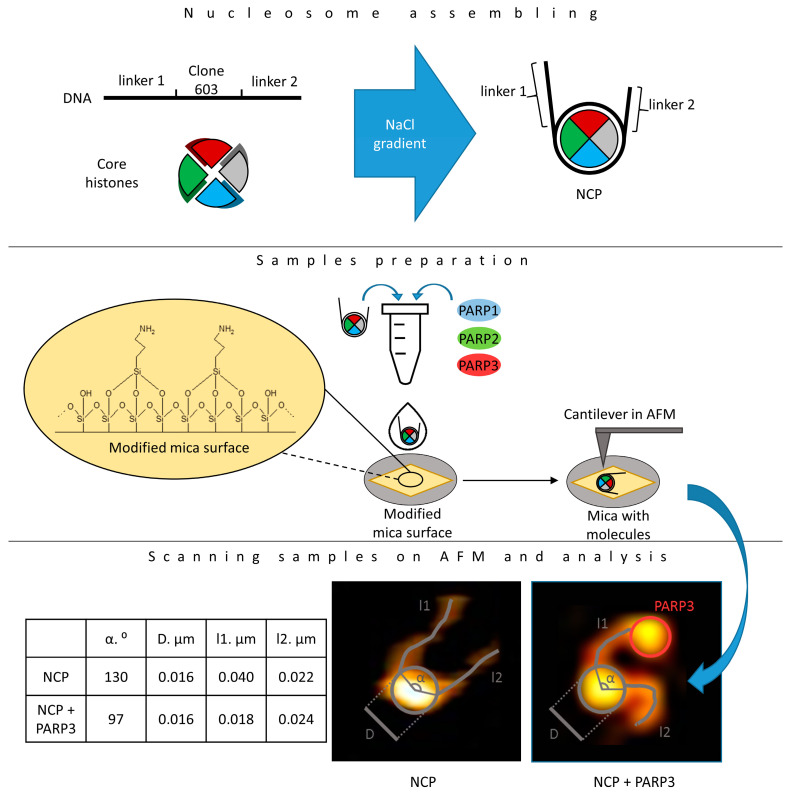
The workflow.

**Figure A2:**
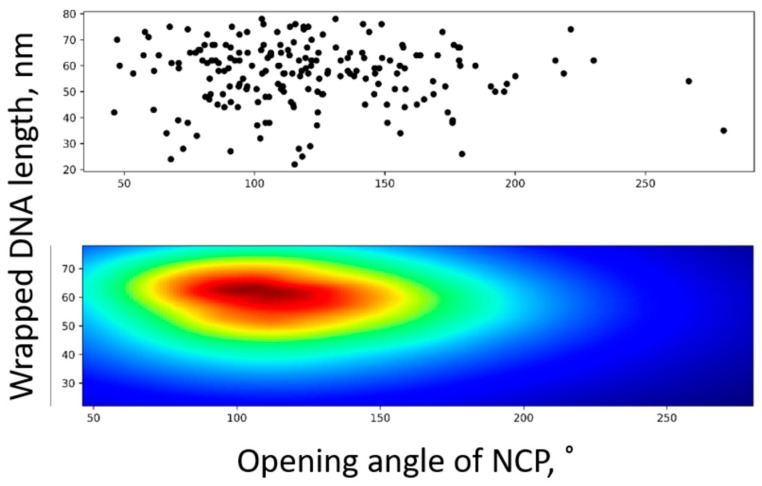
The 2D density plot of wrapped length versus the opening-angle distribution of NCPs.

All images were first processed in the Gwyddion software (http://gwyddion.net/, accessed on 1 March 2023). The ImageJ software (https://imagej.nih.gov/ij/, accessed on 1 March 2023) was employed to measure parameters of the NCP core disk and of the NCP core in complex with PARPs, the length of the NCP DNA arm, and the angle between DNA arms. The arm length was estimated by measuring the DNA from the end point to the point of “entry” into the NCP disk. Diameters of the core and core PARP were estimated as the maximal distance between two parallel tangents to the disk. The angle between NCP DNA arms was defined as an angle formed by two beams from the center of the core disc to the “entry” points of DNA arms. On the basis of the obtained data, histograms and graphs were constructed using the SigmaPlot software v.11.0 (Systat Software Inc., Chicago, IL, USA). Variances in measured values were calculated by means of Student’s *t* distribution with 95% confidence intervals. Measured values are shown diagrammatically in Figure 2a. When sorting PARP–NCP complexes, we chose a distance of 3 nm between the NCP core and a PARP as the border point. The resolution of the cantilever used in this work allowed us to uniquely identify a PARP separated from the NCP core when the distance was more than 3 nm. Therefore, when the proteins were located closer to the NCP core, we assumed that they were directly interacting. The workflow is illustrated in Figure A1.

## Data Availability

The data needed to reproduce our results are contained within the article. Raw data are available upon request.

## Appendix A

**Table A1:** NCP parameters.

NCP #	α, °	D, µm	l1, µm,	l2, µm
1	230	0.011	0.025	0.03
2	59	0.015	0.031	0.015
3	103	0.011	0.032	0.017
4	121	0.016	0.04	0.026
5	128	0.018	0.035	0.024
6	172	0.013	0.029	0.015
7	267	0.011	0.042	0.021
8	106	0.019	0.041	0.028
9	66	0.025	0.057	0.026
10	148	0.021	0.045	0.019
11	124	0.021	0.05	0.03
12	83	0.021	0.045	0.023
13	180	0.016	0.045	0.046
14	136	0.021	0.042	0.017
15	94	0.019	0.039	0.025
16	94	0.018	0.045	0.028
17	68	0.011	0.057	0.036
18	46	0.018	0.037	0.038
19	96	0.023	0.04	0.022
20	169	0.024	0.047	0.021
21	109	0.026	0.029	0.028
22	88	0.017	0.039	0.034
23	111	0.021	0.037	0.03
24	148	0.022	0.033	0.025
25	71	0.021	0.032	0.026
26	280	0.016	0.056	0.026
27	191	0.024	0.041	0.024
28	124	0.021	0.037	0.018
29	102	0.018	0.047	0.038
30	68	0.02	0.035	0.021
31	115	0.022	0.025	0.023
32	90	0.02	0.038	0.027
33	77	0.024	0.033	0.019
34	143	0.02	0.034	0.028
35	162	0.017	0.045	0.027
36	112	0.018	0.04	0.02
37	125	0.016	0.044	0.018
38	124	0.018	0.042	0.025
39	109	0.02	0.028	0.016
40	126	0.023	0.04	0.028
41	74	0.017	0.045	0.034
42	113	0.022	0.024	0.027
43	104	0.02	0.046	0.023
44	121	0.02	0.061	0.027
45	173	0.02	0.043	0.022
46	104	0.017	0.029	0.03
47	133	0.019	0.041	0.014
48	174	0.019	0.043	0.032
49	109	0.015	0.042	0.022
50	83	0.015	0.038	0.024
51	142	0.015	0.052	0.02
52	124	0.018	0.037	0.038
53	179	0.023	0.032	0.018
54	111	0.016	0.045	0.02
55	85	0.02	0.025	0.024
56	115	0.017	0.041	0.032
57	196	0.022	0.043	0.024
58	115	0.018	0.059	0.036
59	97	0.016	0.041	0.025
60	101	0.023	0.048	0.032
61	73	0.016	0.058	0.031
62	67	0.023	0.024	0.018
63	91	0.022	0.055	0.035
64	91	0.027	0.032	0.018
65	126	0.015	0.037	0.031
66	151	0.018	0.046	0.033
67	104	0.015	0.04	0.039
68	78	0.018	0.053	0.031
69	192	0.016	0.045	0.022
70	106	0.017	0.032	0.021
71	119	0.019	0.027	0.015
72	79	0.022	0.039	0.012
73	97	0.018	0.028	0.016
74	120	0.02	0.028	0.022
75	128	0.017	0.036	0.024
76	121	0.019	0.037	0.023
77	131	0.02	0.024	0.015
78	131	0.019	0.036	0.014
79	91	0.014	0.025	0.017
80	146	0.017	0.037	0.021
81	136	0.017	0.031	0.023
82	149	0.019	0.022	0.019
83	141	0.015	0.034	0.018
84	94	0.023	0.027	0.018
85	93	0.016	0.031	0.024
86	110	0.019	0.033	0.016
87	142	0.015	0.029	0.012
88	157	0.014	0.035	0.015
89	117	0.018	0.042	0.047
90	178	0.014	0.032	0.023
91	200	0.014	0.043	0.018
92	106	0.02	0.06	0.019
93	88	0.02	0.035	0.024
94	103	0.02	0.026	0.015
95	109	0.013	0.031	0.026
96	103	0.012	0.035	0.016
97	101	0.012	0.046	0.025
98	118	0.009	0.066	0.026
99	106	0.011	0.033	0.019
100	116	0.013	0.026	0.015
101	158	0.014	0.038	0.035
102	146	0.016	0.046	0.022
103	91	0.015	0.035	0.036
104	97	0.019	0.044	0.021
105	164	0.014	0.029	0.024
106	139	0.017	0.038	0.021
107	197	0.015	0.04	0.024
108	117	0.013	0.037	0.024
109	120	0.018	0.033	0.021
110	75	0.015	0.032	0.02
111	105	0.016	0.04	0.014
112	101	0.015	0.039	0.027
113	178	0.018	0.031	0.019
114	61	0.018	0.034	0.025
115	138	0.013	0.036	0.025
116	91	0.015	0.032	0.022
117	157	0.013	0.027	0.022
118	63	0.015	0.032	0.021
119	144	0.013	0.032	0.012
120	165	0.015	0.051	0.019
121	151	0.018	0.051	0.021
122	86	0.016	0.042	0.014
123	122	0.016	0.039	0.018
124	185	0.016	0.033	0.024
125	110	0.013	0.04	0.012
126	156	0.013	0.038	0.019
127	176	0.017	0.03	0.019
128	103	0.016	0.02	0.019
129	74	0.015	0.024	0.019
130	219	0.019	0.047	0.013
131	97	0.017	0.037	0.015
132	154	0.016	0.042	0.024
133	48	0.015	0.037	0.02
134	114	0.018	0.047	0.024
135	83	0.013	0.041	0.029
136	111	0.018	0.036	0.024
137	86	0.018	0.032	0.027
138	115	0.016	0.052	0.02
139	113	0.017	0.038	0.015
140	94	0.016	0.033	0.019
141	61	0.017	0.04	0.034
142	156	0.016	0.051	0.032
143	71	0.019	0.048	0.03
144	90	0.021	0.04	0.018
145	47	0.018	0.025	0.022
146	84	0.023	0.032	0.017
147	114	0.014	0.032	0.028
148	146	0.016	0.033	0.027
149	78	0.02	0.03	0.021
150	86	0.012	0.043	0.029
151	88	0.016	0.042	0.026
152	99	0.023	0.037	0.02
153	106	0.02	0.031	0.022
154	149	0.02	0.041	0.013
155	110	0.013	0.039	0.027
156	158	0.014	0.038	0.028
157	94	0.016	0.035	0.02
158	80	0.018	0.033	0.022
159	81	0.017	0.042	0.027
160	85	0.011	0.038	0.017
161	58	0.02	0.026	0.018
162	82	0.02	0.034	0.022
163	84	0.017	0.033	0.022
164	158	0.019	0.035	0.023
165	117	0.017	0.028	0.027
166	57	0.015	0.033	0.02
167	111	0.02	0.04	0.025
168	122	0.02	0.039	0.016
169	179	0.019	0.034	0.023
170	221	0.021	0.023	0.02
171	81	0.018	0.031	0.018
172	53	0.017	0.039	0.021
173	94	0.015	0.04	0.025
174	215	0.017	0.038	0.017
175	118	0.018	0.037	0.022
176	176	0.017	0.061	0.017
177	122	0.017	0.028	0.019
178	84	0.02	0.033	0.022
179	155	0.018	0.034	0.026
180	176	0.022	0.053	0.026
181	126	0.02	0.025	0.02
182	89	0.017	0.028	0.021
183	133	0.02	0.041	0.02
184	152	0.016	0.033	0.022
185	122	0.016	0.032	0.023
186	148	0.017	0.039	0.025
187	119	0.014	0.029	0.014
188	82	0.017	0.031	0.014
189	142	0.018	0.032	0.022
190	114	0.017	0.031	0.011
191	170	0.02	0.027	0.026
192	137	0.013	0.04	0.02
193	71	0.018	0.036	0.02
194	103	0.012	0.037	0.023
195	121	0.017	0.024	0.018
196	137	0.015	0.026	0.026
197	125	0.015	0.033	0.019
198	162	0.015	0.028	0.025
199	168	0.016	0.042	0.021
200	120	0.012	0.039	0.019

**Table A2:** NCP parameters in the presence of PARP1.

NCP #	α, °	D, µm	l1, µm,	l2, µm
1	70	0.019	0.053	0.032
2	61	0.029	0	0.027
3	128	0.026	0	0.02
4	100	0.019	0	0.014
5	61	0.023	0.031	0.03
6	96	0.024	0.035	0.019
7	117	0.032	0	0.022
8	97	0.022	0	0.016
9	109	0.023	0	0.019
10	112	0.027	0	0.026
11	120	0.027	0.016	0.033
12	107	0.033	0.023	0.035
13	179	0.023	0	0.032
14	89	0.026	0	0.018
15	58	0.026	0.03	0.021
16	176	0.024	0.019	0.017
17	112	0.024	0.008	0.019
18	112	0.019	0	0.019
19	152	0.018	0.037	0.011
20	99	0.021	0.039	0
21	98	0.022	0	0.02
22	85	0.031	0.012	0.019
23	107	0.02	0	0.024
24	107	0.022	0.033	0
25	68	0.024	0.021	0.018
26	56	0.023	0.023	0.018
27	129	0.023	0	0.02
28	97	0.026	0.008	0.032
29	131	0.023	0.04	0.02
30	85	0.02	0	0.023
31	135	0.024	0.052	0.005
32	118	0.019	0	0.028
33	162	0.024	0.003	0.023
34	107	0.027	0	0.021
35	173	0.031	0	0.022
36	122	0.024	0.04	0
37	103	0.024	0.036	0.025
38	118	0.03	0	0.024
39	98	0.025	0	0.024
40	105	0.026	0	0.024
41	161	0.023	0.044	0
42	121	0.023	0	0.02
43	114	0.023	0	0.024
44	109	0.022	0	0.025
45	85	0.032	0	0.023
46	105	0.035	0.006	0.023
47	93	0.027	0	0.021
48	96	0.023	0	0.023
49	152	0.028	0.015	0.023
50	121	0.021	0	0.015
51	116	0.032	0.031	0.004
52	153	0.024	0.015	0.02
53	87	0.023	0.031	0.008
54	74	0.03	0	0.018
55	67	0.037	0.011	0.019
56	77	0.022	0	0.02
57	76	0.021	0	0.02
58	76	0.026	0	0.02
59	0	0.025	0	0.025
60	116	0.03	0	0.019
61	101	0.033	0.035	0
62	96	0.029	0	0.019
63	145	0.028	0	0.018
64	138	0.027	0	0.027
65	101	0.024	0.022	0.019
66	108	0.023	0.016	0.015
67	220	0.02	0.044	0
68	103	0.026	0	0.02
69	102	0.024	0	0.018
70	129	0.023	0	0.021
71	84	0.018	0	0.021
72	92	0.018	0	0.016
73	98	0.022	0.016	0.023
74	147	0.017	0	0
75	82	0.028	0	0.017
76	130	0.02	0	0.016
77	179	0.029	0.043	0
78	110	0.027	0.043	0
79	130	0.026	0.042	0
80	96	0.019	0.008	0.01
81	83	0.027	0.013	0.024
82	55	0.026	0.038	0
83	144	0.021	0.041	0
84	173	0.026	0	0.018
85	149	0.02	0	0.023
86	164	0.024	0	0.028
87	69	0.026	0	0.022
88	104	0.027	0.013	0.024
89	174	0.02	0	0.023
90	171	0.024	0	0.026
91	106	0.025	0	0.011
92	83	0.021	0	0.018
93	103	0.025	0	0.016
94	120	0.024	0.022	0.021
95	200	0.023	0.033	0.024
96	140	0.023	0	0.022
97	112	0.024	0	0.019
98	168	0.021	0.016	0.02
99	132	0.019	0	0.02
100	112	0.028	0	0.016
101	128	0.024	0.037	0
102	81	0.022	0	0.018
103	63	0.025	0	0.02
104	102	0.026	0	0
105	108	0.021	0.038	0
106	91	0.021	0.039	0
107	84	0.023	0	0
108	93	0.018	0	0.014
109	141	0.021	0	0.013
110	112	0.018	0	0.025
111	75	0.02	0.019	0.019
112	91	0.018	0	0.018
113	66	0.021	0	0.022
114	93	0.026	0	0.018
115	120	0.017	0	0
116	119	0.017	0	0.029
117	142	0.022	0	0.018
118	64	0.021	0	0.026
119	78	0.018	0	0.027
120	82	0.017	0.011	0.028
121	119	0.017	0	0.033
122	85	0.018	0	0.026
123	113	0.02	0	0.025
124	138	0.022	0	0.033
125	140	0.022	0.019	0
126	117	0.017	0.012	0.02
127	97	0.02	0.017	0.025
128	87	0.016	0	0.03
129	157	0.029	0.025	0.025
130	104	0.018	0	0.023
131	63	0.025	0	0.027
132	115	0.023	0.038	0.027
133	133	0.024	0	0.026
134	98	0.029	0	0.023
135	154	0.02	0.009	0.021
136	126	0.022	0.043	0
137	59	0.018	0.037	0.008
138	133	0.018	0	0.019
139	110	0.02	0	0.02
140	132	0.016	0	0.02
141	116	0.02	0.037	0.015
142	124	0.025	0.034	0
143	98	0.014	0.043	0
144	124	0.021	0	0.023
145	111	0.022	0	0.023
146	82	0.022	0.023	0.014
147	173	0.024	0	0
148	141	0.023	0.012	0.026
149	102	0.019	0	0.026
150	115	0.021	0.044	0.009
151	122	0.021	0.007	0
152	85	0.019	0	0.025
153	97	0.021	0	0.027
154	110	0.024	0	0.022
155	137	0.022	0	0.028
156	151	0.021	0.04	0
157	92	0.026	0.04	0
158	112	0.022	0	0.023
159	54	0.014	0.035	0
160	180	0.021	0.014	0.028
161	117	0.021	0	0.018
162	77	0.022	0	0.023
163	116	0.012	0.035	0.007
164	142	0.011	0.03	0.019
165	128	0.015	0.04	0
166	75	0.012	0	0.021
167	178	0.013	0.009	0.005
168	144	0.014	0.035	0
169	155	0.015	0	0.026
170	130	0.014	0.018	0.023
171	120	0.015	0.008	0.024
172	116	0.015	0.014	0.022
173	96	0.014	0.005	0.03
174	151	0.014	0.008	0.033
175	147	0.013	0.043	0
176	69	0.019	0	0.028
177	122	0.021	0.041	0
178	122	0.019	0.036	0
179	151	0.016	0.012	0.019
180	136	0.014	0	0.023
181	148	0.016	0.037	0
182	89	0.014	0.035	0
183	173	0.014	0.009	0
184	162	0.019	0	0.025
185	120	0.021	0	0.019
186	119	0.019	0.039	0
187	85	0.024	0	0.025
188	124	0.017	0.041	0
189	118	0.017	0	0.026
190	150	0.018	0.013	0.024
191	115	0.017	0.037	0
192	90	0.017	0.033	0
193	166	0.017	0	0.028
194	108	0.02	0.035	0
195	161	0.019	0	0.028
196	171	0.016	0	0.025
197	114	0.02	0	0.017
198	87	0.019	0.037	0
199	145	0.016	0	0.027
200	125	0.02	0.011	0.028
201	110	0.021	0	0.028
202	108	0.022	0	0.018
203	74	0.014	0	0.024
204	66	0.018	0.014	0.01
205	92	0.024	0.027	0.025

**Table A3:** NCP parameters in the presence of PARP2.

NCP #	α, °	D, µm	l1, µm,	l2, µm
1	61	0.033	0	0.018
2	144	0.029	0.029	0
3	159	0.028	0.032	0.03
4	60	0.023	0.024	0.016
5	99	0.02	0	0.022
6	105	0.02	0	0.018
7	140	0.024	0	0.02
8	78	0.019	0.042	0.009
9	109	0.021	0	0
10	113	0.018	0	0.023
11	88	0.026	0.023	0.019
12	82	0.019	0	0.022
13	97	0.02	0	0.022
14	185	0.02	0	0.014
15	167	0.019	0	0.021
16	104	0.021	0	0.019
17	77	0.019	0	0.024
18	63	0.022	0	0.022
19	147	0.02	0	0.021
20	108	0.02	0.015	0
21	281	0.018	0	0.029
22	60	0.032	0	0
23	118	0.02	0	0.023
24	140	0.022	0.034	0
25	86	0.019	0	0.024
26	139	0.016	0	0.02
27	88	0.017	0	0.02
28	72	0.019	0.042	0
29	172	0.017	0	0.025
30	160	0.023	0	0.001
31	130	0.024	0.047	0.019
32	94	0.027	0	0.023
33	147	0.027	0.015	0.022
34	174	0.021	0.041	0
35	148	0.025	0	0
36	92	0.02	0.038	0.01
37	146	0.018	0.01	0.029
38	85	0.018	0	0.024
39	98	0.022	0	0.019
40	81	0.017	0.036	0
41	160	0.02	0.04	0.021
42	142	0.021	0	0.024
43	129	0.025	0.007	0.022
44	105	0.022	0.016	0.019
45	134	0.02	0	0.023
46	121	0.023	0.037	0
47	113	0.015	0	0.025
48	170	0.02	0.032	0
49	159	0.025	0.047	0
50	74	0.022	0	0.026
51	132	0.028	0	0.019
52	112	0.015	0	0.023
53	87	0.019	0	0.023
54	124	0.022	0	0.018
55	99	0.022	0	0.021
56	92	0.02	0	0.028
57	93	0.019	0	0.023
58	83	0.016	0	0
59	99	0.022	0	0.023
60	125	0.019	0	0.017
61	114	0.016	0.011	0.018
62	137	0.015	0	0.02
63	126	0.016	0.009	0.023
64	114	0.016	0.037	0
65	104	0.019	0	0.017
66	112	0.022	0	0.025
67	168	0.018	0	0.022
68	102	0.018	0	0.025
69	177	0.027	0.035	0.022
70	105	0.021	0	0.024
71	109	0.023	0	0.023
72	95	0.021	0.034	0.023
73	135	0.018	0.04	0
74	86	0.018	0	0.025
75	95	0.016	0	0.022
76	192	0.016	0	0.023
77	159	0.02	0	0.024
78	116	0.018	0	0.024
79	113	0.019	0.037	0
80	149	0.022	0.023	0.025
81	159	0.021	0	0.022
82	67	0.022	0	0.014
83	130	0.019	0	0.024
84	145	0.02	0.018	0.03
85	95	0.021	0	0.021
86	142	0.018	0.043	0
87	129	0.019	0	0.023
88	216	0.022	0	0.019
89	135	0.02	0	0.033
90	112	0.028	0.04	0.017
91	128	0.019	0.039	0.017
92	117	0.021	0.012	0.024
93	114	0.018	0.044	0
94	115	0.018	0	0.027
95	109	0.022	0.02	0.022
96	112	0.017	0	0.023
97	88	0.02	0.039	0
98	117	0.02	0.015	0.024
99	139	0.018	0	0.024
100	125	0.028	0	0.019
101	95	0.023	0.036	0
102	166	0.031	0.033	0.031
103	94	0.025	0.035	0.004
104	96	0.017	0.014	0.025
105	107	0.023	0	0.021
106	138	0.02	0.015	0.021
107	88	0.016	0	0.008
108	124	0.02	0.035	0
109	90	0.016	0	0.027
110	55	0.014	0.039	0.008
111	134	0.018	0.004	0.019
112	88	0.019	0	0.027
113	77	0.014	0	0.021
114	104	0.019	0.038	0
115	100	0.02	0.033	0.024
116	171	0.017	0	0.024
117	111	0.014	0.024	0.018
118	174	0.019	0.032	0.031
119	136	0.017	0	0.028
120	95	0.017	0.008	0.011
121	174	0.018	0.036	0
122	83	0.019	0.029	0.021
123	127	0.019	0.012	0.022
124	134	0.02	0	0.028
125	146	0.02	0	0.028
126	73	0.018	0.039	0
127	113	0.016	0	0.015
128	111	0.022	0.042	0.016
129	135	0.014	0	0.026
130	128	0.016	0.042	0
131	96	0.017	0.009	0.012
132	156	0.018	0	0.03
133	157	0.019	0	0.029
134	214	0.021	0.009	0.027
135	168	0.019	0.04	0
136	99	0.017	0	0.029
137	83	0.02	0	0.024
138	123	0.015	0.036	0
139	114	0.017	0	0.032
140	121	0.021	0	0.028
141	144	0.018	0	0.025
142	97	0.018	0.04	0
143	143	0.018	0.036	0
144	104	0.02	0.038	0.019
145	99	0.018	0	0.027
146	85	0.019	0	0.02
147	103	0.017	0	0.027
148	143	0.017	0.045	0
149	133	0.016	0	0.032
150	111	0.016	0.006	0.029
151	153	0.019	0.013	0.03
152	109	0.019	0.008	0.028
153	128	0.014	0	0.032
154	93	0.021	0.007	0.029
155	99	0.02	0.04	0.01
156	120	0.017	0.035	0.007
157	96	0.017	0.038	0.002
158	111	0.014	0.038	0
159	122	0.017	0.011	0.028
160	133	0.014	0.008	0.028
161	113	0.021	0	0.028
162	73	0.015	0.004	0.03
163	113	0.018	0.01	0.032
164	97	0.019	0.04	0
165	86	0.017	0.038	0
166	142	0.017	0.007	0.029
167	77	0.019	0.007	0.028
168	78	0.018	0.007	0.019
169	154	0.015	0	0.027
170	128	0.02	0	0.031
171	125	0.02	0.042	0
172	101	0.018	0.01	0.023
173	109	0.017	0.037	0
174	86	0.017	0	0.026
175	103	0.015	0	0.031
176	142	0.02	0.01	0
177	102	0.02	0	0.029
178	152	0.018	0	0.027
179	172	0.016	0	0.025
180	118	0.019	0	0.028
181	148	0.019	0	0.03
182	102	0.016	0	0.026
183	84	0.021	0.037	0.013
184	145	0.015	0	0.031
185	95	0.014	0	0.024
186	144	0.015	0.033	0
187	102	0.02	0.007	0.018
188	217	0.016	0.048	0
189	172	0.015	0	0
190	145	0.018	0.04	0
191	162	0.018	0.006	0
192	107	0.022	0.005	0.025
193	123	0.014	0.041	0
194	84	0.018	0.037	0
195	267	0.019	0.049	0.029
196	114	0.017	0.039	0
197	96	0.016	0	0.024
198	91	0.019	0	0.023
199	148	0.017	0	0.008
200	136	0.019	0.033	0.005
201	85	0.019	0.039	0

**Table A4:** NCP parameters in the presence of PARP3.

NCP #	α, °	D, µm	l1, µm,	l2, µm
1	106	0.025	0.005	0.025
2	156	0.024	0	0.024
3	87	0.02	0	0.02
4	77	0.024	0	0.025
5	98	0.025	0	0.022
6	77	0.015	0	0.027
7	81	0.017	0	0.024
8	138	0.02	0.041	0.01
9	83	0.018	0	0.016
10	102	0.021	0.035	0.009
11	93	0.016	0	0.027
12	95	0.013	0.035	0
13	119	0.019	0	0.021
14	174	0.021	0	0.021
15	122	0.017	0	0.023
16	97	0.013	0.04	0
17	84	0.018	0.037	0
18	75	0.02	0.043	0
19	84	0.024	0	0.024
20	96	0.018	0	0.028
21	145	0.015	0	0.023
22	69	0.02	0	0.024
23	81	0.018	0	0.028
24	103	0.018	0	0.021
25	79	0.018	0.041	0
26	154	0.019	0	0.032
27	99	0.018	0	0.023
28	106	0.015	0	0.024
29	116	0.018	0	0.023
30	87	0.021	0	0
31	98	0.018	0.041	0
32	134	0.019	0	0.021
33	111	0.02	0.038	0
34	104	0.018	0	0.026
35	161	0.016	0	0.023
36	109	0.017	0	0.027
37	114	0.015	0	0.028
38	110	0.018	0	0.027
39	116	0.019	0	0.021
40	101	0.014	0.039	0
41	75	0.012	0	0.023
42	135	0.018	0.034	0
43	83	0.017	0	0.023
44	94	0.014	0	0.02
45	87	0.019	0	0.023
46	157	0.016	0.042	0.015
47	81	0.019	0	0.03
48	62	0.02	0	0.032
49	95	0.02	0	0.022
50	132	0.018	0.038	0
51	108	0.017	0.038	0
52	71	0.015	0	0.024
53	140	0.017	0	0.021
54	99	0.019	0	0.014
55	45	0.017	0	0.024
56	94	0.019	0	0.024
57	119	0.014	0	0.023
58	116	0.018	0	0.021
59	38	0.019	0	0.018
60	74	0.016	0	0.023
61	53	0.015	0.037	0
62	95	0.018	0	0.02
63	166	0.014	0.015	0.021
64	61	0.015	0	0.019
65	122	0.018	0	0.024
66	209	0.016	0	0.023
67	119	0.015	0	0.023
68	78	0.014	0	0.023
69	121	0.012	0.037	0
70	118	0.017	0	0
71	101	0.017	0.04	0.01
72	105	0.018	0.027	0.022
73	87	0.016	0.009	0.024
74	66	0.016	0	0.018
75	178	0.017	0.038	0.014
76	161	0.026	0.016	0.023
77	97	0.016	0.018	0.024
78	82	0.018	0	0.024
79	142	0.016	0.035	0
80	123	0.016	0	0.026
81	82	0.015	0	0.023
82	133	0.013	0	0.022
83	71	0.017	0	0.024
84	93	0.019	0.008	0.022
85	105	0.021	0	0.024
86	98	0.016	0	0.028
87	144	0.014	0	0.028
88	113	0.02	0.007	0.021
89	124	0.015	0	0.027
90	107	0.016	0	0.022
91	85	0.016	0	0.026
92	154	0.018	0.011	0.028
93	119	0.015	0	0.019
94	90	0.019	0	0.021
95	106	0.018	0	0.024
96	87	0.017	0.04	0
97	60	0.017	0.038	0.011
98	83	0.014	0.038	0
99	119	0.017	0	0.022
100	118	0.014	0	0.023
101	89	0.014	0	0.025
102	99	0.016	0.004	0.025
103	131	0.017	0	0.02
104	118	0.015	0	0.021
105	102	0.015	0	0
106	68	0.015	0.009	0.025
107	86	0.013	0	0.026
108	82	0.016	0.017	0.022
109	104	0.016	0	0.022
110	119	0.015	0.039	0.004
111	74	0.013	0.01	0.026
112	100	0.017	0.008	0.003
113	96	0.017	0	0.02
114	89	0.012	0.038	0
115	117	0.015	0.038	0
116	105	0.014	0	0.024
117	59	0.016	0	0.028
118	151	0.013	0	0.03
119	115	0.013	0.005	0.028
120	83	0.017	0	0.006
121	169	0.014	0.019	0.031
122	130	0.02	0.043	0.004
123	135	0.015	0.034	0
124	110	0.012	0.008	0.017
125	122	0.016	0	0.026
126	105	0.019	0.013	0
127	96	0.014	0.012	0.02
128	93	0.018	0	0.026
129	132	0.018	0	0.027
130	85	0.018	0	0.024
131	85	0.015	0	0.03
132	93	0.016	0.008	0.026
133	99	0.02	0	0.028
134	97	0.015	0.039	0
135	123	0.02	0	0.021
136	95	0.013	0	0.029
137	97	0.018	0.04	0.006
138	119	0.014	0.038	0
139	93	0.016	0	0.024
140	93	0.017	0.013	0.017
141	97	0.017	0.033	0.006
142	109	0.017	0	0.023
143	98	0.016	0	0.021
144	113	0.022	0.026	0.027
145	79	0.018	0	0.026
146	134	0.023	0	0
147	117	0.021	0	0.026
148	60	0.016	0.035	0
149	86	0.02	0.009	0.024
150	111	0.019	0.034	0
151	148	0.018	0	0.024
152	45	0.017	0	0.031
153	92	0.017	0.038	0
154	93	0.017	0	0.024
155	99	0.021	0.042	0.015
156	93	0.022	0	0.031
157	114	0.018	0.015	0.028
158	99	0.023	0.019	0.027
159	89	0.018	0.008	0.022
160	140	0.021	0	0.034
161	87	0.017	0.016	0.025
162	94	0.019	0.037	0
163	108	0.02	0	0.025
164	89	0.02	0.032	0
165	86	0.022	0.006	0.028
166	100	0.021	0.009	0.029
167	75	0.019	0.038	0
168	52	0.02	0.037	0
169	114	0.018	0	0.027
170	97	0.018	0.04	0
171	91	0.017	0.013	0.024
172	81	0.021	0.045	0
173	152	0.027	0.031	0
174	91	0.018	0	0.028
175	170	0.019	0.017	0.033
176	55	0.02	0.006	0.024
177	88	0.017	0	0.026
178	81	0.023	0	0.029
179	59	0.018	0	0
180	138	0.017	0.037	0
181	74	0.017	0	0.023
182	75	0.019	0.036	0
183	82	0.02	0	0.024
184	102	0.023	0.016	0.004
185	170	0.02	0.044	0
186	113	0.017	0	0.023
187	71	0.021	0.039	0
188	157	0.019	0	0.027
189	156	0.018	0.038	0
190	170	0.018	0	0.006
191	85	0.022	0.042	0.006
192	104	0.015	0	0.021
193	175	0.021	0.04	0.009
194	117	0.019	0.038	0.014
195	56	0.018	0.042	0
196	126	0.015	0	0.027
197	99	0.019	0	0.032
198	96	0.015	0	0.022
199	98	0.018	0.009	0.023
200	65	0.021	0.042	0
201	70	0.026	0	0.029
